# Predictors of adherence of enhanced external counterpulsation in patients with coronary heart disease after discharge: A mixed-methods study

**DOI:** 10.3389/fcvm.2022.1005958

**Published:** 2022-11-25

**Authors:** Yuhuan Yin, Qinli He, Rong Zhang, Hu Cheng, Yiyin Zhang, Juxia Zhang

**Affiliations:** ^1^School of Nursing, Gansu University of Chinese Medicine, Lanzhou, China; ^2^Department of Cardiology, Gansu Provincial Hospital, Lanzhou, China; ^3^Clinical Educational Department, Gansu Provincial Hospital, Lanzhou, China

**Keywords:** EECP, coronary heart disease, discharge, adherence, factors

## Abstract

**Background:**

Although enhanced external counter pulsation (EECP) has been included in the cardiac rehabilitation prescription for coronary heart disease (CHD) in China, because the total treatment duration of a course of EECP is 36–36 h, the average hospital stay of CHD patients is short, and the adherence after discharge remains unclear. The purpose of this study is to investigate the adherence to EECP in CHD patients after discharge, and analyze the related influencing factors.

**Methods:**

A retrospective mixed method study combining qualitative and quantitative methods. Quantitative component included CHD patients who had received EECP treatment between March 2020 and August 2021. The qualitative component included in-depth interviews with patients who did not adhere to EECP after discharge. Binary Logistic regression was used to analyze the predictors of EECP adherence after discharge. In-depth interviews with patients were conducted to explore the reasons for dropping out of the EECP after discharge.

**Results:**

Among 1,304 patients, only 24.23% adhered to EECP treatment after discharge. Binary logistic regression results showed that patients with disease duration < 2 years (OR = 3.13, 95%CI: 2.31–4.24), high school or below (OR = 2.81, 95%CI: 1.98–4.01), distance between residence and hospital more than 20km (OR = 2.08, 95%CI: 1.47–2.96), age over 60 (OR = 2.00, 95%CI: 1.46–2.74), female (OR = 1.64, 95%CI: 1.78–2.29), and angina pectoris (OR = 1.65, 95%CI: 1.16–2.34) were more likely to not adhere to EECP treatment after discharge. However, patients with monthly family income over 8000¥ (OR = 0.46, 95%CI: 0.28–0.75) were more likely to adhere to EECP treatment after discharge than those with household monthly income below 4,000¥. In the qualitative results, the reasons why patients do not adhere to EECP after discharge mainly include insufficient understanding, restricted objective conditions and psychosocial factors.

**Conclusions:**

The adherence of CHD patients to EECP treatment after discharge was poor. It is necessary to develop effective intervention measures, such as brochures or videos to improve patients' understanding of the importance of adherence to EECP treatment after discharge. In addition, offering EECP treatment during off-hours and weekends may also improve adherence in more young patients.

## Introduction

Cardiovascular disease is the main cause of death worldwide, among which coronary heart disease (CHD) has the highest morbidity and mortality, posing a serious threat to human health (1). As the secondary prevention of cardiovascular diseases, exercise-centered cardiac rehabilitation is an important part of medical care for all patients with heart disease (2). However, about a third of patients have severe lifestyle restrictions that prevent them from exercising (3), which increases the dropout rate from cardiac rehabilitation to some extent. Meta-analysis showed that the drop-out rate for enrolled patients in high-income countries was 12-56% (4). The dropout rate in Iran was reported to be as high as 82% (5). A survey of 283 patients after PCI in China found that the dropout rate of exercise training program was 36.44% (6).

Enhanced external counterpulsation (EECP) is a non-invasive, safe and cost-effective adjunctive therapy approved by the US Food and Drug Administration (7). It can not only be used as an alternative therapy in addition to drugs and surgical treatment of cardiovascular diseases, but also has been included in the prescription of cardiac rehabilitation in China (8). A large number of clinical randomized controlled trials have proven that EECP can improve myocardial ischemia and hypoxia and left ventricular function, reduce angina attacks, and improve exercise tolerance (9–11). Long-term adherence to EECP treatment can significantly improve the endothelial function of patients, thereby inhibiting the occurrence and development of atherosclerotic lesions (12). Moreover, EECP is considered to be a “passive” exercise and thus can significantly benefit patients who are unable to exercise (13).

The expert consensus on EECP treatment in China suggested the course of EECP is 1h per day, and the total duration of a course is 35–36 h (14). However, the average length of hospital stay for CHD patients is about 3–7 days, which requires that most patients to come to the hospital for treatment every day after discharge. EECP has been developed in China for more than 50 years, but it was introduced in Gansu Province in 2020, CHD patients' adherence to EECP treatment after discharge remains unclear. Therefore, the purpose of this study is to investigate the adherence to EECP treatment in CHD patients after discharge, and analyze the related influencing factors, in order to provide reference for healthcare workers to formulate related measures.

## Methods

### Study design and population

This was a mixed method study with qualitative and quantitative components. The quantitative component was a retrospective cohort analysis based on the medical and rehabilitation records of CHD patients who received EECP treatment during their hospitalization in Gansu Provincial Hospital from March 1, 2020 to August 31, 2021. Inclusion criteria for the retrospective cohort study: (1) Patients who received 5-10h of EECP treatment during hospitalization from March 1, 2020 to August 31, 2021; (2) Patients diagnosed with CHD by coronary angiography; (3) Age 18 years or older.

Adherence criteria for EECP after discharge: The latest expert consensus in China recommends that the course of EECP is 1 h a day, with a total course of 35–36 h. For statistical analysis, we defined the adherence rate as at least 70% (25 h) based on the actual EECP treatment of our patients, which was considered as the patients' adherence to EECP.

The qualitative component was in-depth interviews with key informants who did not adhere to EECP treatment after discharge. We conducted a purposeful sampling, and identified key interviewees based on the sociodemographic characteristics of patients, disease characteristics and geographical location. Because patients had been discharged and were geographically widespread, 19 respondents were selected for semi-structured telephone interviews. To ensure consistency of the collected data, all interviews were conducted by the principal investigator (YYH). The interview consisted of three core questions: “How do you feel after EECP treatment? Has it relieved your symptoms?”, “Do you know what benefits EECP can bring to your health?” and “What made you unwilling to adhere to EECP treatment after discharge?”. Participants were encouraged to explain and elaborate on their answers.

### Data collection and procedure

#### Quantitative data

the hostipal numbers of patients receiving EECP treatment was queried through the rehabilitation notebook, and then the baseline characteristics of patients were queried through the electronic medical record system according to the hospital numbers. Baseline characteristics included: (1) Sociodemographic characteristics: sex, age, educational level, monthly family income, health insurance, distance from residence to hospital, BMI, smoking; (2) Clinical characteristics: course of disease, angina pectoris, hypertension and history of PCI surgery. The researchers input the relevant information into the Microsoft Excel, which was double-checked by two researchers.

#### Qualitative data

All interviews were recorded. After the interview, the researchers transcribed the entire recording word by word, input the original qualitative data into Microsoft Word, and then sorted out, analyzed and classified according to topics and questions to determine common topics.

### Data analysis

#### Quantitative data

SPSS 21.0 was used for statistical analysis of quantitative data. First, frequencies (n) and proportions (%) were used to describe the general characteristics (sociodemographic characteristics and clinical characteristics) in adherence group and non-adherence group. Second, chi-square test and was performed to preliminarily analyse general characteristics related to EECP adherence after discharge (yes/no). Finally, binary logistic regression was used to examine the independent factors associated with EECP adherence after discharge. The dependent variable was EECP adherence treatment after discharge (adherence=0, non-adherence = 1), with the significant factors in univariate analyses included as independent variables. Odd ratio (OR) with 95% confidence intervals (95%CI) were used as the measure of association. OR = 1 indicates that there was no correlation between exposure factors and outcome (EECP non-adherence); OR>1 indicates that exposure factors are risk factors for EECP non-adherence; OR < 1 indicates that exposure factors are protective factors for EECP non-adherence. The statistical tests were two-sided, and the effects with *p* < 0.05 were considered to be statistically significant.

#### Qualitative data

NVivo version 12.0 (QSR International) was used for data transcription and analyzed by thematic analysis. Data were subject to thematic analysis using the framework approach (15). YYH (who has 2 years of qualitative research training) and HQL(teacher of qualitative research training in Department of Cardiology, has 8 years of qualitative research experience) read all recordings carefully and repeatedly to extract relevant statements and meanings, and then look for common conceptual or meaningful features to form topic groups and categories, with topics associated with the raw data. The obtained data were analyzed, summarized and classified. ZJX (who has 2 years of qualitative research training and 6 years of qualitative research experience) reviewed the analysis to reduce bias and increase the credibility of the interpretation.

### Ethics approval

This study was approved by the Ethics Committee of Gansu Provincial Hospital (2021-234). The quantitative component was a retrospective analysis of the basic characteristics of the patients, so informed consent was not required. Informed consent was obtained for key interviewees in the qualitative component. All clinical investigations were conducted according to the principles expressed in the Declaration of Helsinki.

## Results

### Quantitative results

#### Patients' characteristics

Among 1,304 patients, only 24.23% adhered to EECP treatment after discharge. 62.12% were male and 61.81% had lower education. Only 13.88% of patients had a monthly income of more than 8,000¥, and more than 70% lived more than 20 km away from the hospital (Table 1).

**Table 1:** Demographic characteristics of coronary heart disease patients.

**Characteristics**	**Overall patients (*N =* 1,304), *n* (%)**	**Adherence (*N =* 316), *n* (%)**	**Non-adherence (*N =* 988), *n* (%)**	***p***
Gender				< 0.001^*^
Male	810 (62.12)	244 (77.22)	566 (57.29)	
Female	494 (37.88)	72 (22.78)	422 (42.71)	
Age (years)				< 0.001^*^
< 60	730 (55.98)	206 (65.19)	524 (53.04)	
≥60	574 (44.02)	110 (34.81)	464 (46.96)	
Educational level				< 0.001^*^
High school or below (≤ 12 years)	830 (61.81)	114 (36.08)	716 (72.47)	
College degree or above (≥15 years)	474 (38.19)	202 (63.92)	272 (27.53)	
Monthly family income (¥)				< 0.001^*^
< 4,000	566 (43.40)	82 (25.95)	484 (48.99)	
4,000–8,000	564 (43.25)	135 (42.72)	429 (43.42)	
>8,000	174 (13.34)	99 (31.33)	75 (7.59)	
Medical insurance				< 0.001^*^
Medical insurance for urban workers	550 (42.18)	209 (66.14)	341 (34.51)	
Medical insurance for urban residents	224 (17.18)	46 (14.56)	178 (18.02)	
New rural cooperative	380 (29.14)	45 (14.24)	335 (33.91)	
Self pay	150 (11.50)	16 (5.06)	134 (13.56)	
Distance (km)				< 0.001^*^
>20	958 (73.47)	166 (52.53)	792 (80.16)	
≤ 20	346 (26.53)	150 (47.47)	196 (19.84)	
BMI (kg/m^2^)	24.79 ± 3.29	24.92 ± 3.54	24.74 ± 3.20	0.408
Course of disease (year)				< 0.001^*^
< 2	864 (66.26)	134 (42.41)	730 (73.89)	
≥2	440 (33.74)	182 (57.59)	258 (26.11)	
Smoking				0.881
No	986 (75.61)	240 (75.95)	746 (75.51)	
Yes	318 (24.39)	76 (24.05)	242 (24.49)	
Angina pectoris				< 0.001^*^
No	922 (70.71)	248 (78.48)	674 (68.22)	
Yes	382 (29.29)	68 (21.52)	314 (31.78)	
Post-PCI				< 0.001^*^
No	1088 (83.44)	216 (68.35)	872 (88.26)	
Yes	216 (16.56)	100 (31.65)	116 (11.74)	
Hypertension				0.246
No	656 (50.31)	168 (53.16)	488 (49.39)	
Yes	648 (49.69)	148 (46.84)	500 (50.61)	

PCI, percutaneous coronary intervention.

Data are reported as n (%) unless otherwise noted; Age data are presented as mean±SD.

* p < 0.01.

#### Factors associated with EECP treatment after discharge

The results of univariate analysis showed that patients with different gender, age, education level, family monthly income, medical insurance, distance, course of disease, angina pectoris and Post-PCI had statistically significant differences in their adherence to EECP after discharge (Table 1).

Binary logistic regression results in Table 2 showed that patients with disease duration < 2 years (OR = 3.13, 95%CI: 2.31–4.24), high school or below (OR = 2.81, 95%CI: 1.98–4.01), distance between residence and hospital more than 20 km (OR = 2.08, 95%CI: 1.47–2.96), age over 60 (OR = 2.00, 95%CI: 1.46–2.74), female (OR = 1.64, 95%CI: 1.78–2.29), and angina pectoris (OR = 1.65, 95%CI: 1.16–2.34) were more likely to not adhere to EECP treatment after discharge. However, patients with monthly family income over 8,000¥ (OR = 0.46, 95%CI: 0.28–0.75) were more likely to adhere to EECP treatment after discharge than those with household monthly income below 4,000¥.

**Table 2:** Factors associated with EECP adherence after discharge.

**Factors**	**OR (95% CI)**	***p***
Gender (female)	1.64 (1.78–2.29)	0.003^*^
Age (≥60 years)	2.00 (1.46–2.74)	< 0.001^*^
Educational level (high school or below)	2.81 (1.98–4.01)	< 0.001^*^
Monthly family income (¥)
< 4,000		
4,000–8,000	1.04 (0.72–1.52)	0.830
>8,000	0.46 (0.28–0.75)	0.002^*^
Medical insurance
Medical insurance for urban workers		
Medical insurance for urban residents	0.97 (0.62–1.50)	0.879
New rural cooperative	1.26 (0.82–1.92)	0.295
Self pay	1.79 (0.97–3.29)	0.062
Distance (>20 km)	2.08 (1.47–2.96)	< 0.001^*^
Course of disease (≤ 2 years)	3.13 (2.31–4.24)	< 0.001^*^
Angina pectoris (yes)	1.65 (1.16–2.34)	0.005^*^
Post-PCI (yes)	0.73 (0.51–1.05)	0.093

Dependent variable assignment: adherence = 0, non-adherence = 1.

OR, odds ratio; CI, confidence interval; PCI, percutaneous coronary intervention; Ref, Reference group.

* p < 0.01.

### Qualitative results

#### Sample characteristics

A total of 19 patients were interviewed, including 11 females and 8 males. The specific characteristics were shown in Table 3.

**Table 3:** General characteristics of participants in the qualitative study.

**Characteristics**	**All interviews (*N =* 19), *n* (%)/$\bar{\text{x}}$±SD (range)**
Age (years)	61.33 ± 11.27 (40-81)
Educational level
Junior high or below	7 (36.84)
High school	5 (26.32)
College or above	7 (36.84)
Occupation
Farmers	6 (31.58)
Employed	7 (36.84)
Retiree	6 (31.58)
Financial support
No	5 (26.32)
Yes	14 (73.68)
Distance (km)
≤ 20	9 (47.37)
>20	10 (52.63)
Course of disease (years)
< 2	9 (47.37)
≥2	10 (52.63)
Angina pectoris
No	12 (63.16)
Yes	7 (36.84)
History of surgery
No	12 (63.16)
PCI	5 (26.32)
CABG	2 (10.53)

PCI, percutaneous coronary intervention; CABG, Coronary Artery Bypass Graft.

#### Barriers to adherence to EECP treatment after discharge

**Figure 1:**
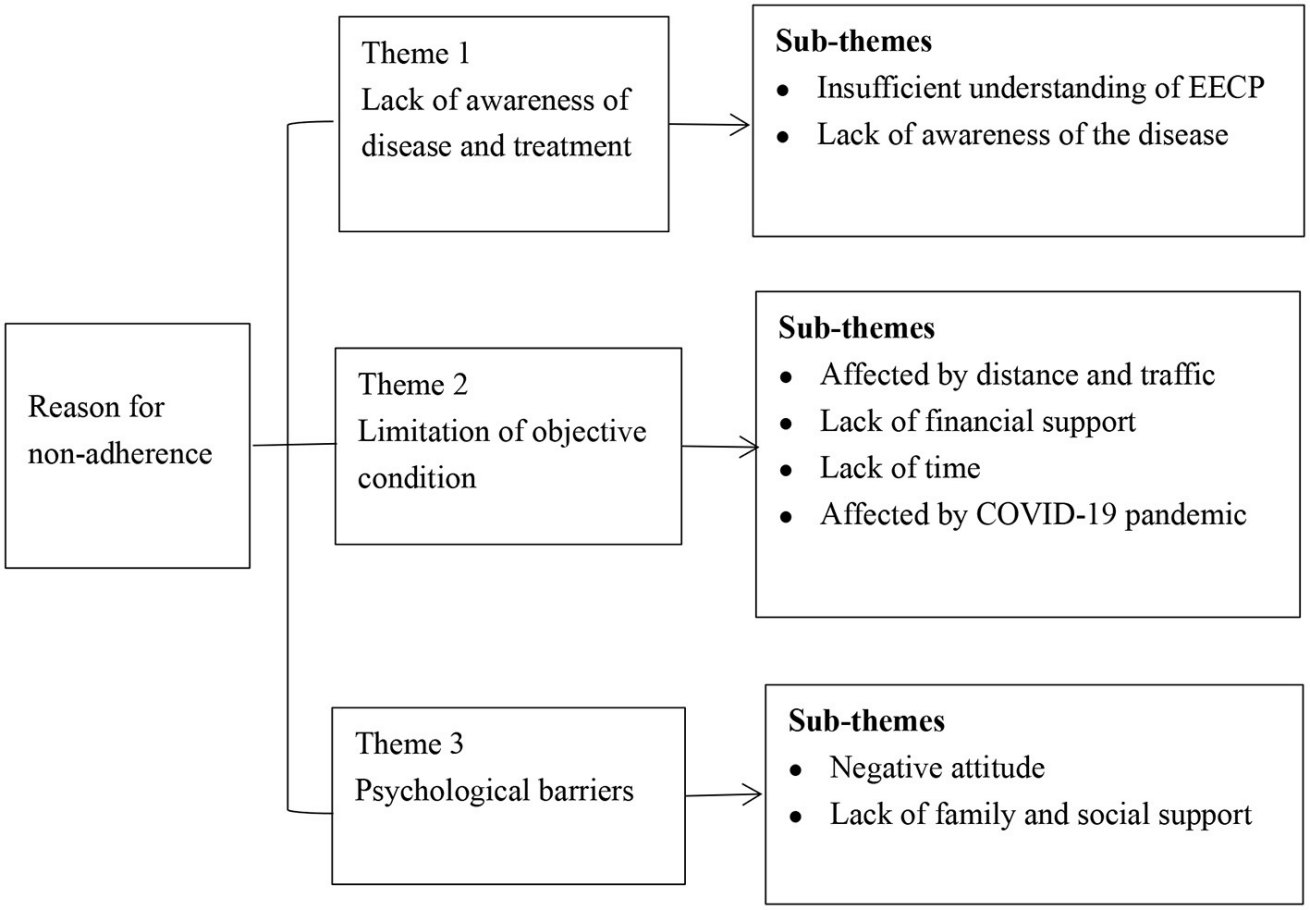
Diagrammatic of the themes and subthemes within the thematic framework.

The qualitative results showed that the most important reasons for CHD patients not to adhere to EECP after discharge were insufficient understanding of the efficacy of EECP, distance and transportation, and lack of financial support. In addition, COVID-19, lack of time, negative attitude and lack of awareness of the disease also hinder patients' adherence to EECP treatment after discharge (Figure 1 and Table 4).

**Table 4:** Analysis of causes and representative quotes of non-adherence to EECP in CHD patients after discharge.

**Themes**	**Subthemes**	**Representative quotes**
Lack of awareness of disease and treatment	Insufficient understanding of EECP	“*I'm a farmer, I do farm work at home, so these exercises don't do much for me”* “*I have been hospitalized three times this year and each time I have been treated with EECP, but I never recovered. I think it useless to do so”* “*EECP is indeed beneficial, but I feel much better after discharge, so I don't think it is necessary to continue treatment”*
	Lack of awareness of the disease	“*Only an operation can cure my disease”* “*I have too many physical diseases, and I think my disease can only be slowly maintained by medication”*
Limitation of objective condition	Affected by distance and traffic	“*Our home is too far from the hospital to come back every day for treatment”* “*My house is not far from the hospital, but I can't drive, so it is not convenient to go to and from the hospital”*
	Lack of financial support	“*I also want to continue to adhere to EECP treatment, but I have no money. My economic situation is so poor that I have spent so much money in hospitalization. If I come to the hospital for treatment every day after discharge, it will be covered by outpatient reimbursement. The outpatient reimbursement rate is too low, which will cost too much”*. “*The total duration of a course of EECP is too long and costly”*
	Lack of time	“*I know that this EECP has a good effect on my disease, but I have no time to hospital for treatment after discharge. I need to work, and the outpatient department of the hospital closes after I get off work in the evening, so I have no choice but to give up the rest of the course”* “*I have to take care of the elderly and children in my family, so I don't have time to go to the hospital for treatment every day”*
	Affected by the COVID-19 pandemic	“*I got out of the hospital just as the COVID-19 was getting worse, and I had to give up”*
Psychological barriers	Negative attitude	“*My chest pain has been incurable and has affected my life so much that I don't want treatment. I think I have an incurable disease”* “*I have an incurable disease, and I don't want any more treatment, any more burden on my family”*
	Lack of family and social support	“*I did this EECP treatment during the hospital, which was useful for the improvement of my symptoms. After discharge, my family did not allow me to continue to do this treatment”*

#### Insufficient understanding of EECP

The reason why most patients did not adhere to EECP treatment after discharge was due to insufficient understanding of the efficacy of EECP, especially older and less educated patients. Most patients were unaware of the benefits of adherence to EECP treatment.

“*I'm a farmer, I do farm work at home, so these exercises don't do much for me”*. [Male, 68 years]

“*I have been hospitalized three times this year and each time I have been treated with EECP, but I never recovered. I think it useless to do so”* [Male, 71 years]

However, there were also some patients who believed that although EECP has brought benefits to their health, they thought their disease was much better after discharge than before hospitalization, so there was no need to continue treatment.

“*EECP is indeed beneficial, but I feel much better after discharge, so I don't think it is necessary to continue treatment”*. [Female, 63 years]

#### Affected by distance and traffic

As EECP was carried out in provincial hospitals, some patients in remote areas cannot continue to receive EECP treatment after discharge due to distance and transportation.

“*Our home is too far from the hospital to come back every day for treatment”*. [Male, 51 years]

#### Lack of financial support

More than half of patients lacked financial support and considered that the 35-day course was too long and expensive.

“*I also want to continue to adhere to EECP treatment, but I have no money. My economic situation is so poor that I have spent so much money in hospitalization. If I come to the hospital for treatment every day after discharge, it will be covered by outpatient reimbursement. The outpatient reimbursement rate is too low, which will cost too much”*. [Female, 60 years]

In addition to the above three important factors, failure to adhere to EECP after discharge was also associated with the COVID-19, negative attitude, time and the awareness of disease.

“*I got out of the hospital just as the COVID-19 was getting worse, and I had to give up”*. [Male, 62 years]

“*I know that this EECP has a good effect on my disease, but I have no time to hospital for treatment after discharge. I need to work, and the outpatient department of the hospital closes after I get off work in the evening, so I have no choice but to give up the rest of the course”*. [Male, 46 years]

Importantly, negative attitude mainly occurred in female and angina patients.

“*My chest pain has been incurable and has affected my life so much that I don't want treatment. I think I have an incurable disease”*. [Female, 52 years]

## Discussion

The results of this study showed that the adherence rate of CHD patients to EECP treatment after discharge was only 24.23%, which was similar to the adherence to exercise training programs in cardiac rehabilitation (16, 17). Studies have proved that adhering to a complete course of EECP treatment can not only improve the cardiac function of CHD patients, relieve the onset of angina, but also improve their sleep, anxiety, depression, and thus improve the quality of life (18–21). However, the dropout rate of CHD patients after discharge was quite high in this study. If patients only receive EECP treatment during hospitalization and do not adhere to EECP after discharge, the total duration of EECP treatment is too short, the expected therapeutic effect may not be achieved.

The results of this study showed that education level and disease duration were the two strongest predictors of the adherence of EECP treatment after discharge, which was consistent with Parashar et al.'s findings (22). Importantly, Rosengren et al. showed that education level is also one of the major risks factor for cardiovascular diseases (23). People with lower levels of education may be less aware of the importance of seeking timely care or have reduced access to information on how to access care and overcome existing barriers (23). These patients may often not understand the benefits of EECP for their health or believe that these health problems can be self-managed (4, 24). In the qualitative results, we found that insufficient understanding of the efficacy of EECP was the main reason for patients not to adhere to EECP treatment after discharge. Most of these patients were older and had lower education level, they believed that EECP was no different from exercise. Therefore, patients who could exercise alone had poor adherence after discharge. Meanwhile, some patients have a false perception that they are getting better after discharge and therefore there is no need to continue treatment, especially newly diagnosed patients. However, with longer time to diagnosis, patients' perceived risk of disease may increase, as well as access to knowledge and programs about cardiac rehabilitation.

Studies found that female patients were more likely to have multiple comorbidities, or believe that their disease was at high risk and that such participation was futile (25, 26), so this perception may be a barrier for patients to adhere to EECP treatment after discharge. Meanwhile, other studies have found that women were more likely to have problems with transportation and family responsibilities (27, 28). Interestingly, in the qualitative results we found that due to the limitations of disease, lack of financial resources and family support, female patients were more likely to have negative emotions, which became an important barrier to adhere to EECP treatment after discharge. Huffman et al. found that optimism and positive emotions can improve patients' adherence to cardiac rehabilitation (29). Therefore, medical workers should pay more attention to the patients' psychological problems, encourage caregivers to provide more family support. Meanwhile, nurses can also encourage patients with good prognosis to share their experiences and the importance of completing 35 h course of EECP, in order to relieve these patient' negative emotions and improve their confidence in disease treatment, thus helping to improve the adherence to EECP treatment after discharge.

In previous studies, distance has been reported as a common factor affecting adherence to cardiac rehabilitation (26, 30, 31). Our study showed that patients who live more than 20 km away from the hospital have poor adherence, which may be related to the fact that EECP treatment is carried out in provincial grade A hospitals, while most patients come from rural areas or other remote areas. Qualitative results showed that even though patients were aware of the importance of EECP adherence after discharge, they could not accept the time and expense of daily trips to and from the hospital, and as the cost of each EECP treatment is 75¥, which exceeds the expected cost of patients. Meanwhile, the reimbursement rate of treatment expenses after discharge is low, which greatly affects patients' adherence to EECP treatment. Therefore, the availability of EECP treatment in smaller tertiary hospitals or community centers and increased health insurance reimbursement rates will help improve patients' adherence to EECP treatment after discharge.

However, we found that patients with angina pectoris had poor adherence after discharge. Previous studies found that patients with angina pectoris were often hindered from participating in exercise training programs due to pain and limited activity, resulting in a higher dropout rate (32, 33). Importantly, in the qualitative results, we also found that patients with angina were more likely to have negative attitude, they had a wrong understanding that angina pectoris is an incurable disease, such practical issues need to be considered. In fact, they did not know that 35 h course of EECP has been shown to relieve angina attacks and reduce the use of nitroglycerin, which may also be related to a lack of physician advice and encouragement (34, 35). The intensity of physician encouragement was reported to be a key factor in determining patients to participate and adhere to cardiac rehabilitation (36). This may also be due to the fact that EECP treatment has only been carried out in Gansu province in the past 2 years, and there was still a lack of understanding and knowledge of EECP among healthcare workers, so the information transmission to patients was not enough. In addition, due to the large workload and the fast turnover of patients, the health education for patients was not in place, resulting in the patients did not realize the importance of complete the remaining courses of EECP after discharge. Zhang et al. found that developing brochures or making educational videos for illiterate patients might help patients better understand the program (37). Therefore, it is necessary to formulate practical and effective training and health education to improve patients' understanding of the role of EECP and their motivation to adhere to treatment after discharge. In addition, offering EECP treatment during off-hours and weekends may improve compliance in more young patients.

## Limitations

Our quantitative study was retrospective and did not cover all factors associated with non-adherence to EECP after discharge due to limited information in the medical record database. More prospective, multi-center studies are needed in the future to further explore the factors and obstacles affecting the EECP adherence in patients with coronary heart disease.

## Conclusions

Patients with coronary heart disease had lower adherence to EECP treatment after discharge, especially elderly patients with lower education level. The main barriers include insufficient awareness of the efficacy of EECP, as well as the influence of distance and economic conditions. On the one hand, it is necessary to formulate practical and effective training and health education to improve patients' understanding of the role of EECP and their motivation to adhere to treatment after discharge. On the other hand, the availability of EECP treatment in smaller tertiary hospitals and community centers, with benefits through health insurance, will help improve patients' adherence to EECP treatment after discharge.

## Data availability statement

The original contributions presented in the study are included in the article/supplementary material, further inquiries can be directed to the corresponding author/s.

## Ethics statement

The studies involving human participants were reviewed and approved by the Ethics Committee of Gansu Provincial Hospital. The patients/participants provided their written informed consent to participate in this study.

## Author contributions

The conception and design of the study: JZ, YY, and QH. Acquisition of data: YY, RZ, YZ, and HC. Analysis and interpretation of data: YY, RZ, and HC. Drafting the article: YY and JZ. Revising the article: JZ, YY, YZ, and QH. Funding acquisition: JZ and QH. Final approval of the version to be submitted: all authors.

## Funding

This research was funded by the Natural Science Foundation of Gansu Province (21JR7RA607 and 21JR7RA613), National Natural Science Foundation of China (72264002), the China Medical Education Association Project (2022KTZ010), and Health industry scientific research project of Gansu Province (GSWSKY-2019-50).

## Conflict of interest

The authors declare that the research was conducted in the absence of any commercial or financial relationships that could be construed as a potential conflict of interest.

## Publisher's note

All claims expressed in this article are solely those of the authors and do not necessarily represent those of their affiliated organizations, or those of the publisher, the editors and the reviewers. Any product that may be evaluated in this article, or claim that may be made by its manufacturer, is not guaranteed or endorsed by the publisher.
